# The Structure‐Mechanics Relationship of Bamboo‐Epidermis and Inspired Composite Design by Artificial Intelligence

**DOI:** 10.1002/adma.202414970

**Published:** 2024-12-27

**Authors:** Zhao Qin, Aymeric Pierre Destree

**Affiliations:** ^1^ Laboratory for Multiscale Material Modelling Syracuse University 151L Link Hall Syracuse NY 13244 USA; ^2^ Department of Civil and Environmental Engineering Syracuse University 151L Link Hall Syracuse NY 13244 USA; ^3^ The BioInspired Institute Syracuse University Syracuse NY 13244 USA

**Keywords:** bamboo epidermis, bio‐inspired design, deep convolutional generative adversarial networks, fracture toughness, particle‐reinforced composite

## Abstract

Bamboo culm has been widely used in engineering for its high strength, lightweight, and low cost. Its outermost epidermis is a smooth and dense layer that contains cellulose, silica particles, and stomata and acts as a water and mechanical barrier. Recent experimental studies have shown that the layer has a higher mechanical strength than other inside regions. Still, the mechanism is unclear, especially for how the low silica concentration (<10%) can effectively reinforce the layer and prevent the inner fibers from splitting. Here, theoretical analysis is combined with experimental imaging and 3D printing to investigate the effect of the distribution of silica particles on composite mechanics. The anisotropic partial distribution function of silica particles in bamboo skin yields higher toughness (>10%) than randomly distributed particles. A generative artificial intelligence (AI) model inspired by bamboo epidermis is developed to generate particle‐reinforced composites. Besides the visual similarity, it is found that the samples by the generative model show failure processes and fracture toughness identical to the actual bamboo epidermis. This work reveals the micromechanics of the bamboo epidermis. It illustrates that generative AI can help design bio‐inspired composites of a complex structure that cannot be uniformly represented by a simple building block or optimized around local boundaries. It expands the design space of particle‐reinforced composites for enhanced toughness modulus, offering advantages in industries where mechanical reliability is critical.

## Graphical abstract

This article investigates the micromechanics of bamboo epidermis, focusing on how anisotropic silica particle distributions enhance toughness. By integrating experimental imaging, 3D printing, and generative AI, the study develops bio‐inspired particle‐reinforced composites with mechanical properties akin to bamboo. The findings expand design possibilities for composites, offering innovative solutions for applications demanding high toughness and mechanical reliability.

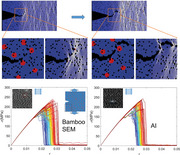

## 1 Introduction

**Figure 1:**
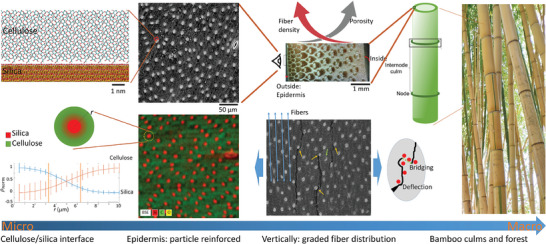
The multiscale structure of the bamboo epidermis. At the macroscale, images show bamboo forests and culms (photo credit to Z.Q.). At the mesoscale, the graded fiber distribution is along the direction of culms, with densely packed fibers of high density on the outside epidermis and loosely packed fibers of low density inside the culm. The orientation of the fibers highly correlates to the direction of crack propagation in the epidermis, which is mainly caused by fiber splitting, as shown in the SEM image. The distribution of silica particles plays a vital role in defecting the crack propagation and forming bridges to increase the ductility and energy release before failure. The fact that these are silica particles with well‐defined sizes and circular shapes embedded in the bamboo epidermis is shown by SEM and BSE images. Based on the current study of the Si/C distribution, we conclude the mean size of silica particles is 10 µm. Former atomistic simulations of the silica‐cellulose interface have shown a higher shear strength than that of each material phase.^[^ ^10^ ^]^

As the stem of the fastest‐growing plant, bamboo culm is a highly sustainable natural material widely used in engineering fields for its high strength/weight ratio (e.g., 3–4 times that of steel).^[^ ^1^, ^2^, ^3^ ^]^ The unidirectional fibers contribute to the bamboo's remarkable axial mechanics (e.g., high elastic modulus and strength in tension, etc.),^[^ ^1^, ^4^ ^]^ enabling them to be used as fencing, furniture, handicrafts, and reinforcing agents in polymer matrices.^[^ ^5^ ^]^ It is hollow for the internode section (**Figure** **1**), making the culm lightweight with an advantage over wood, fiberglass composite, and steel for scaffolding in modern constructions and many engineering applications.^[^ ^2^, ^6^, ^7^ ^]^ Axial compression and lateral loading force on the culm can come from the application of gravity and wind loading force in nature.^[^ ^8^, ^9^ ^]^ Forces over distance along the slender culm lead to significant bending moment and cause the maximum stress in the epidermis layer.^[^ ^10^ ^]^ It is thus attractive how such a layer prevents bamboo from failure in extreme loading force. Besides direct usage, bamboo can be laminated and bonded to form bulk plywood.^[^ ^11^ ^]^ A recent study focusing on the densified bamboo material has shown that the bamboo samples with the epidermis have 40% higher strength, 17% higher Young's modulus, and 12.8% higher thermal conductivity than samples without the epidermis.^[^ ^11^ ^]^ These observations suggest that the epidermis of bamboo is stiffer and more robust than other regions inside the culm.

The mechanical failure of the bamboo epidermis in an extreme loading condition initiates from naturally existing defects at the microscopic scale (e.g., stomata^[^ ^12^, ^13^ ^]^; see the microscopic image of *Pseudosasa amabilis* in Figure 1) that lead to longitudinal fiber splitting at the larger scales. However, this layer is rich in embedded silica particles, which are much stiffer than cellulose matrix and can reinforce this composite material. A former study of the mechanical properties of particle‐reinforced polymer composites has shown that well‐bonded small particles are crucial to enhancing the fracture toughness of the composite.^[^ ^14^ ^]^ The microscopic images of the bamboo epidermis have shown that the typical silica is small (≈10 µm) in diameter (Figure 1; Figure S1, Supporting Information). A recent study of the silica‐cellulose interface of bamboo epidermis has shown that hydrogen bonds fully connect the two material phases at equilibrium and the shear strength reaches 10 GPa at their interface, which is significantly higher than the yield strength of silica (2.5 GPa) or cellulose (600 MPa) per se.^[^ ^10^ ^]^ The result suggests that the small silica particles and cellulose matrix are bonded well, agreeing with the general design principle of particle‐reinforced composites for high toughness. Moreover, there is evidence to show that the particles can delay crack propagation, as is observed in the fractured samples in the microscope (Figure 1).^[^ ^15^ ^]^ Besides the molecular interface, the intrinsically disordered particle distribution adds structural complexity and brings uncertainty to the mechanical modeling of the bamboo epidermis, making it difficult to be uniformly represented by a simple representative building block. Microstructure designs using generative AI represent a transformative approach that is not limited to a fixed building block. By leveraging advanced algorithms, such as Deep Convolutional Generative Adversarial Networks (DCGANs),^[^ ^16^ ^]^ multimodal models that fuse image and language information during learning,^[^ ^17^ ^]^ and multimodal large language models,^[^ ^18^ ^]^ researchers can emulate the intricate microstructural patterns found in nature, such as the anisotropic particle distributions in bamboo or the hierarchical porosity in bone. Generative AI also enables rapid exploration of vast design spaces, creating novel microstructures different from the training input.

**Figure 2:**
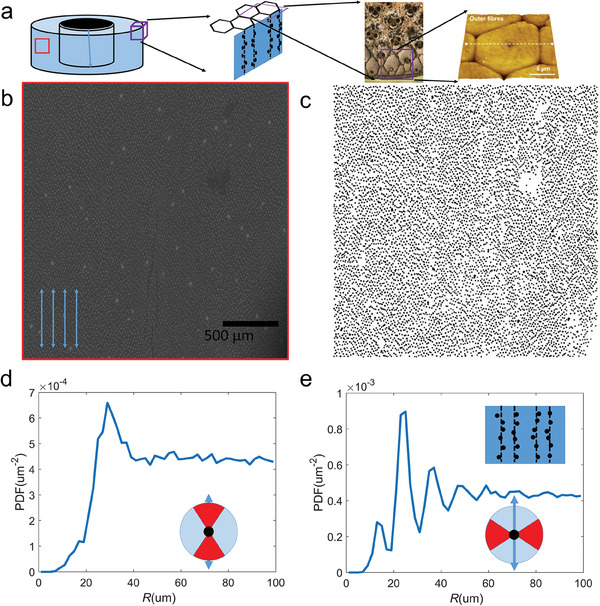
The heterogeneous distribution of silica particles in different directions: along and perpendicular to fibers. a) Schematic image of the bamboo culm, the hexagon arrangement of closely packed fibers near the outside epidermis, an optical microscopic image of the arrangement of bamboo fibers, and an AFM image of the hexagonal topology of the fiber geometry at the cross‐section of the culm near the epidermis.^[^ ^24^ ^]^ The microscopic images are reproduced.^[^ ^24^ ^]^ with copyright. b) A typical SEM image of the bamboo epidermis with particle distribution, with silica particles shown in a light gray color and cellulose matrix in a darker color. The inserted arrows indicate the direction of the fibers as obtained from FFT (see Figure S1, Supporting Information for details), agreeing with the direction of fiber splitting as shown. Scale bar: 500 µm. c) We extracted the location and size for each silica particle in panel B using a Hough transform algorithm (see Method for details), giving an image of silica particle distribution with high contrast. d) The PDF of particle distribution only along the fiber direction ($\frac{\pi }{2}-\frac{\pi }{6}<\varphi <\frac{\pi }{2}+\frac{\pi }{6}$, as defined in Equation (1)). e) The PDF of particle distribution is only vertical to fiber direction ($-\frac{\pi }{6}<\varphi <\frac{\pi }{6}$).

Many pieces of evidence suggest that silica particles reinforce the soft cellulose matrix and prevent the defects from propagating in mechanical loading. How the particle distribution function contributes to the composite mechanics is still being determined. This fundamental question goes beyond the effects of particle size, interfacial interaction, phase mechanics, and volume ratio but focuses on the relationship between the distribution configuration and the composite mechanics. Indeed, it is shown that hard particles at the front of a pre‐existing crack tip can significantly enhance the material strength and fracture toughness, not only for bamboo.^[^ ^10^ ^]^ but other heterogeneous composites.^[^ ^19^ ^]^ Other than the strong periodicity in the direction perpendicular to the fiber orientation, as is revealed by the Fast Fourier Transform (FFT) to the SEM images (Figure S1c, Supporting Information), the distribution of the particles shows overall short‐range order (SRO) but some long‐range order (LRO) in specific directions by referring to the structural orderings in atomic systems.^[^ ^20^ ^]^ The FFT images show a largely diffusive, uniform distribution of intensity and a large brighter ring far from the center in most directions that suggest the SRO feature, except for the several distinct bright spots perpendicular to the fibers in the middle that suggest the LRO feature. We quantify the particle distribution with the SEM images by extracting their coordinate centers through a Hough transformation (Section Supporting Experimental Methods, Supporting Information), and performing partial distribution functions (PDF, Experimental Section) calculation to analyze the spatial particle arrangements (**Figure** **2**; Figure S1d, Supporting Information). The PDF results suggest that the SRO, as indicated by the brighter ring in FFT, is primarily determined by the uniform distance from a particle to its nearest neighbors, and such a uniform distance is efficient in enhancing the composite against randomly appearing cracks.^[^ ^10^ ^]^ However, how LRO contributes to the material strength and toughness has yet to be understood. Literature has shown, for example, that the amorphous polymer domain lack of LRO is crucial to material ductility and energy dissipation^[^ ^21^, ^22^ ^]^; high‐entropy alloys of heterogeneous elemental distribution without LRO have tortuous dislocation path and high toughness.^[^ ^23^ ^]^ It is natural to hypothesize that the silica particle distribution lack of LRO benefits bamboo toughness, but the mechanism is yet to be determined.

## 2 Results and Discussion

### 2.1 Anisotropic Particle Distribution in Bamboo Epidermis

We visualize the distribution of silica particles within the epidermis surface of *Pseudosasa amabilis* bamboo samples with an SEM. According to the back‐scattered electron (BSE) image of SEM, we identify the interface between silica and cellulose at the surface of silica particles (Figure 1). We used Hough's transformation to extract the coordinates of each silica particle center, statistically measure the silicon and carbon distribution around the centers, and thus measure the diameter of silica particles varies from 6.6 to 12.6 µm with a mean value of 10.0 µm (Figure 1; Figure S2, Supporting Information for details). To understand how the particle distribution relates to the microstructure of bamboo (Figure 2a), we extract the coordinate of these particles from epidermis SEM (Figure 2b,c) and carry out directional partial distribution function (PDF) analysis (Figure 2d,e). SRO refers to the consistent and predictable arrangement of particles over short distances, typically involving only nearest neighbors, and is present in both ordered and amorphous structures. In contrast, LRO represents a repeating, periodic arrangement of particles extending over large distances, characteristic of highly ordered structures, and absent in amorphous ones. It is revealed that instead of isotopic distribution, the distribution perpendicular to fibers is of LRD as each center particle has 4–5 clear neighbors away from it (Figure 2e). Still, the distribution along the fibers is only of SRO as each center particle has only one clear neighbor as the distribution lacks of order beyond the first neighbor (Figure 2d; Figure S1e, Supporting Information, suggesting average distance from a particle to its nearest neighbors is 27 ± 7.8 µm). The results are highly repetitive (Figure S1e,f, Supporting Information). They are interesting outcomes as they suggest that the distribution of particles in the bamboo epidermis is neither random nor ordered. Instead, their distribution is anisotropic, with the LRD only perpendicular to the fibers.

The series of sharp PDF peaks are obtained at very consistent positions 12.2 ± 1.1, 22.2 ± 2.3, 33.4 ± 3.3, and 44.2 ± 3.9 µm for the perpendicular‐fiber direction (Statistical results of Figure S1f, Supporting Information). Their overall ratio (≈1:2:3:4) agrees very well with the hexagonal packing of bamboo fiber bundles (Figure 2a) in literature, which points out the fiber diameter near the epidermis is 16.62 ± 3.57 µm,^[^ ^24^ ^]^ this value falls between 1/3 of the sum of the first two peaks (11.5 µm) and 2/3 of the sum of the first two peaks (23.0 µm), suggesting the bamboo epidermis cuts through the hexagonal lattice of the fiber distribution in parallel to the armchair edge (schematic insert of Figure 2e). The results of silica particle distribution, together with the geometry and distribution of fiber bundles beneath the epidermis, suggest that the silica particles are located right around the junction between two neighboring fiber bundles along the bamboo culm.

### 2.2 Bamboo‐Epidermis Mimicking Structures for Mechanical Tests

Because the bamboo fibers are long and highly oriented along the bamboo culm, fiber splitting is the main form of mechanical fracture (Figure 1). It is less understood how the disordered arrangement of silica particles along the fiber direction effectively dissipates the energy during mechanical fracture. To understand their mechanical contribution, we develop 3D printing models by following the geometric information obtained from the SEM images.

**Figure 3:**
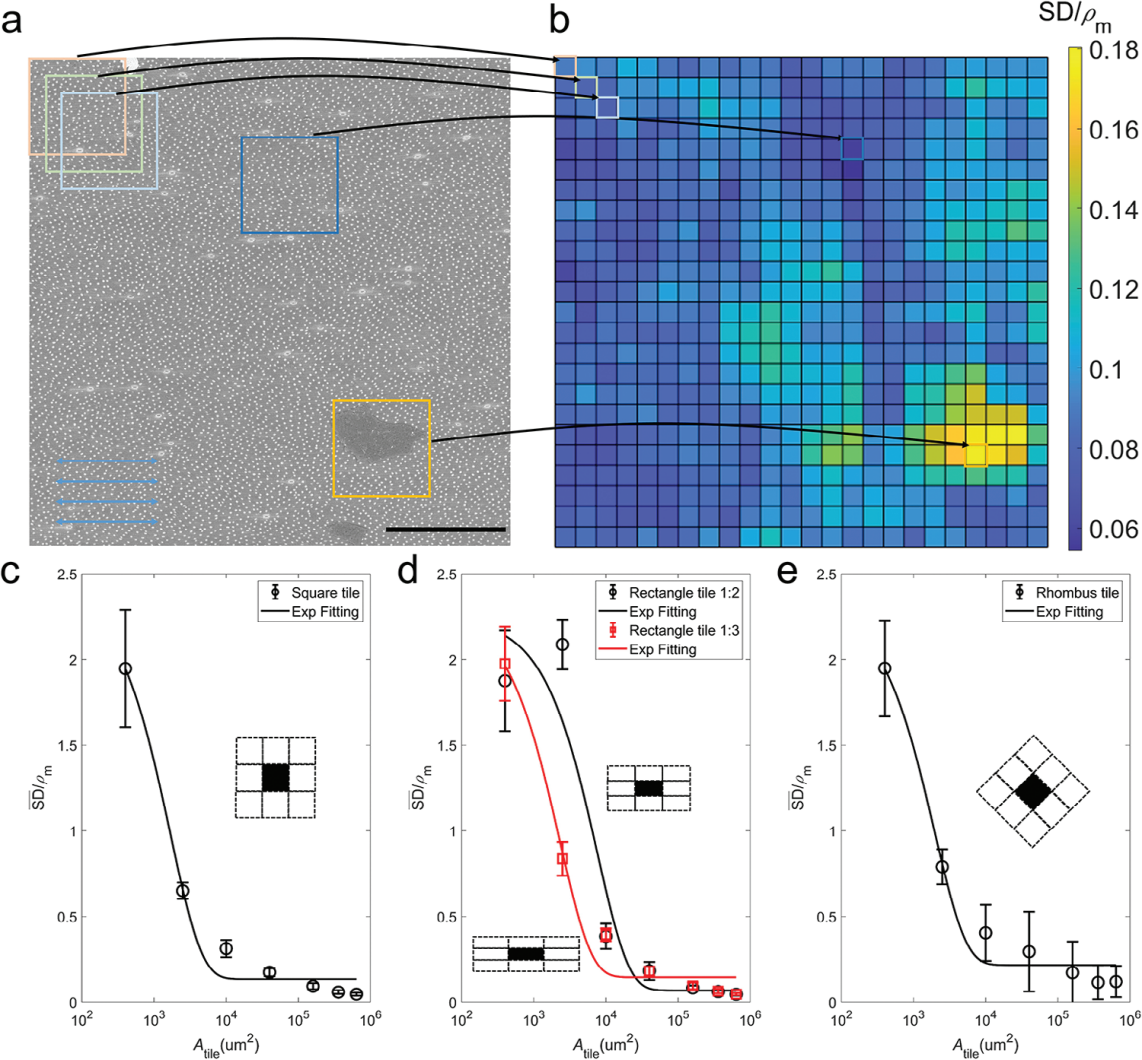
Extracting representative unit cells of different shapes to represent silica particle distribution. a) A SEM image with square unit cells of 400 × 400 µm^2^ taken from different locations. The arrows indicate the direction of the fibers beneath. Scale bar: 500 µm. b) The summary of the normalized deviation of the PDF of the periodic assembly of any selected unit cell from the PDF of the original bamboo sample, given as SD/ρ_*m*_ for unit cells taken from different sample locations (see Experimental Section and Figure S3, Supporting Information for details). The regions with significant structural defects are shown to have large deviations from the original sample. The mean $\bar{\mathrm{SD}}/\rho_{m}$ value of the varying unit area *A_tile_* for c) square units, with a fitting curve of a function $\bar{\mathrm{SD}}/\;\rho_{m}=0.13+2.3e^{-A_{tile}/1700}$; d) rectangular units, with different aspect ratios with fitting curves of functions $\bar{\mathrm{SD}}/\;\rho_{m}=0.07+2.2e^{-A_{tile}/7600}$ and $\bar{\mathrm{SD}}/\;\rho_{m}=0.15+2.2e^{-A_{tile}/2300}$ for 1:2 and 1:3 ratios, respectively; and e) rhombus units, with a fitting curve of a function $\bar{\mathrm{SD}}/\;\rho_{m}=0.2+2.1e^{-A_{tile}/1900}$. It is shown that a larger cell is generally effective in representing the original distribution of silica particles.

We first develop a technique to obtain a representative unit cell from the SEM image and use the cell to design models. **Figure** **3**a,b summarize the difference between the PDF of the periodic assembly of the representative unit cells and that of the original SEM. These results highlight that using a small unit cell makes it hard to fully represent the distribution features (with a minimum deviation, i.e., $\bar{\mathrm{SD}}/\rho_{m}$ value). While a considerable unit cell will inevitably yield a low deviation, it is not helpful as the large unit cell essentially takes the entire original SEM image as the unit cell. We fit the relationship between a unit cell area and the mean deviation value with an exponential decaying function, which reveals the critical cell area (*A*_0_) of a balance between patterns with a low deviation value ($\bar{\mathrm{SD}}/\rho_{m}$) and small unit cell sizes. It is shown that a square unit cell is generally more effective in representing the silica distribution (Figure 3c, *A*_0_ =  1700 µm^2^) than a rectangular unit cell (Figure 3d, *A*_0_ =  7600 and 2300 µm^2^ for aspect ratio of 1:2 and 1:3, respectively) and a rhombus unit cell (Figure 3e, *A*_0_ =  1900 µm^2^). These results suggest that while the unit cell length along the fiber direction for rectangular unit cells is more critical in reducing the deviation, this trend does not hold for square unit cells, which gives more effective usage of unit cell size.

**Figure 4:**
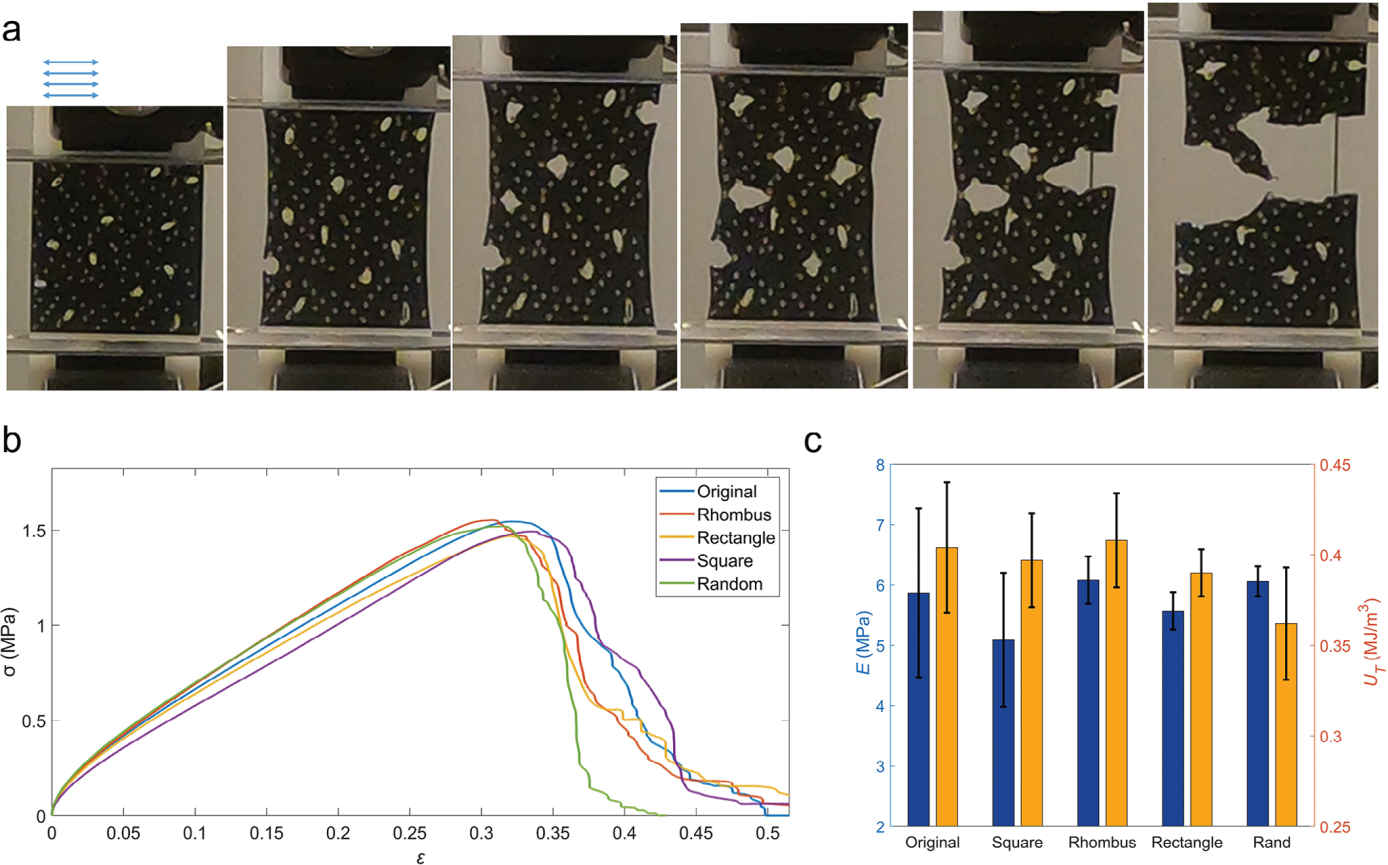
Mechanical performance of bamboo‐epidermis‐inspired composite material. a) Experimental snapshots of a multi‐material 3D printed sample that mimics bamboo epidermis with the rigid material (Digital ABS in white color) for silica particle and soft material (Flexi Black in black color) cellulose matrix. The arrows indicate the direction of the fibers corresponding to the SEM image. Randomly distributed cracks of elliptical shape are initially assigned to the sample to represent stomata that may facilitate crack initiation. It is shown that the rigid particles can deflect the crack propagation and form bridges to enhance sample ductility and toughness. b) The mean stress‐strain (σ − ε) curves (average of 3 samples for each curve) of mechanical samples composed of unit cells of different geometries but of the same area. c) The summary of the Young's modulus (*E*) and toughness modulus (*U_T_*) of samples obtained from different sources.

We compare the mechanics of bamboo epidermis with unit‐cell‐inspired designs. We directly crop a 400 × 400 µm^2^ sample from the SEM image of the original bamboo epidermis and convert that into a multimaterial 3D printing sample for quasi‐static mechanical loading test (**Figure** **4**a, see Experimental Section for printing details and Supporting Information for mechanical testing details). It is shown that the cracks initiate from the structural defects of stomata within the epidermis, while the hard particles delay the crack propagation through crack deflection and bridging. We repeat the mechanical test three times with very similar outcomes and compute the mean stress–strain (*σ−ε*) relationship given in Figure 4b. The printing and tests are repeated for samples made of a periodic assembly of unit cells (40 × 40 µm^2^ for square, 30 × 90 µm^2^ for rectangles and 45 × 45 µm^2^ for rhombus), as well as a 400 × 400 µm^2^ sample of random distribution of particles with their *σ−ε* curves summarized in Figure 4b and the material elastic modulus (*E*) and toughness modulus (*U_T_*) summarized in Figure 4c. It is shown that the toughness modulus of samples based on the distribution of bamboo particles is very similar and over 10% higher than that of purely random distribution (Table S1, Supporting Information). The result agrees with fracture toughness as obtained in the former finite element modeling result.^[^ ^10^ ^]^ However, the former study focuses on the ultimate strength before crack propagation but here we consider the energy release up to the total failure of the sample. We also note that the samples obtained from square and rectangle unit cells are of lower *E* because of a slightly lower ratio of particle volume, which agrees with other particle‐reinforced composites.^[^ ^14^ ^]^

### 2.3 DCGAN for Automatic Generation of Bamboo‐Epidermis‐Inspired Structures with Similar Mechanics

Although the unit cells can represent the mechanics of the bamboo epidermis in tensile loading, they face several limitations. First is the need for more variability and adaptability because the uniformity of the repeating unit imposes limitations on the range of possible forms and functions. Moreover, it needs to be clarified how to tune the mechanics by varying the position of the particles within the unit cell and, therefore, respond to diverse loading conditions or complex geometry. For example, a long pre‐existing crack taking place along the boundary between unit cells may find significantly less resistance for its propagation than cracks through the unit cells. There are inconsistent results, as we have seen in our fracture test on 3D‐printed samples with a long pre‐existing crack.

**Figure 5:**
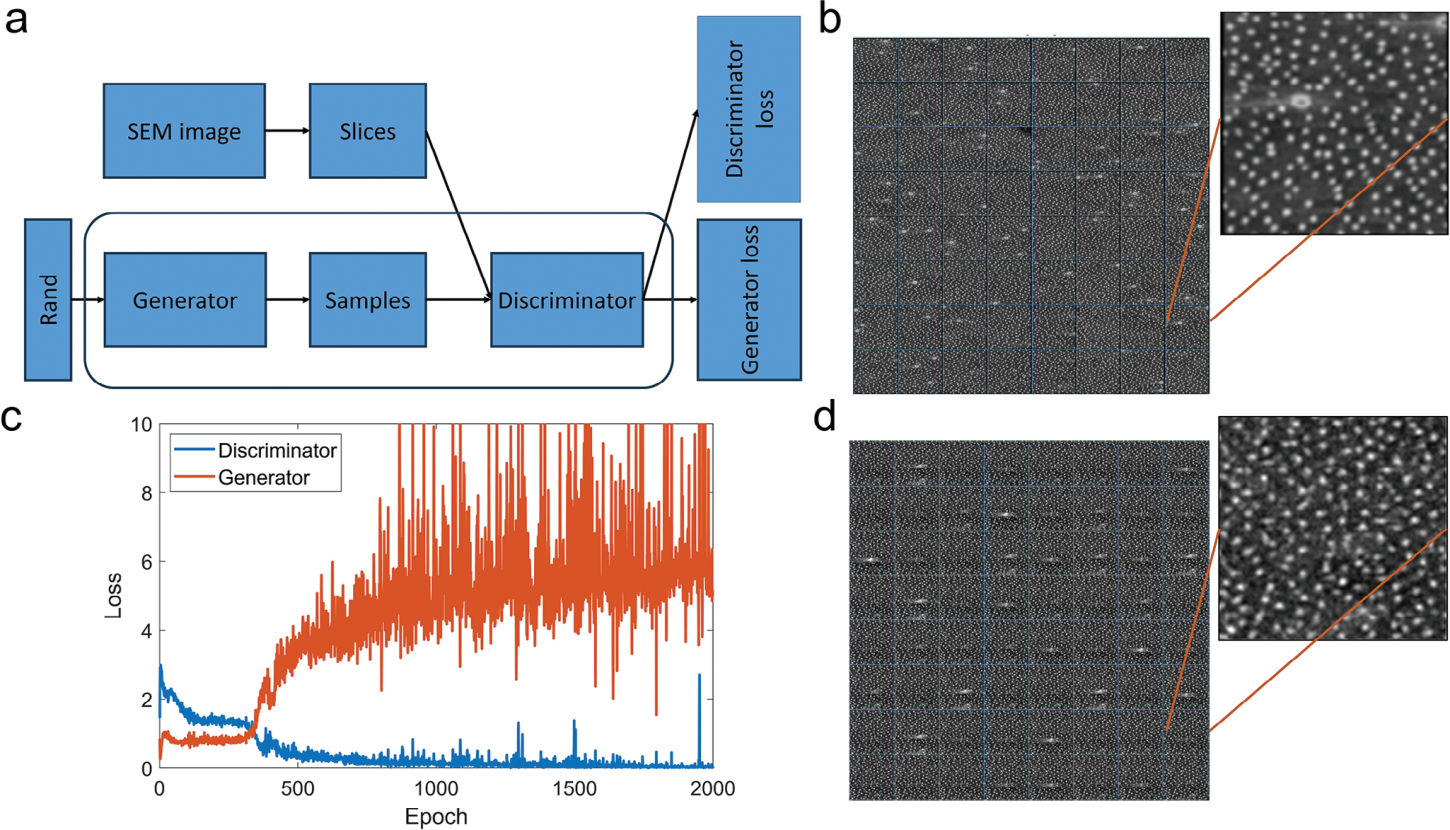
Using DCGAN to learn the intrinsic hidden patterns from the bamboo epidermis images and generate synthetic ones. a) The general workflow of DCGAN is composed of two neural networks: a generator and a discriminator that compete and coevolve during the training. b) The example is 64 slices of SEM images of the bamboo epidermis (each corresponds to 400 × 400 µm^2^ physical size). c) The log loss function for the discriminator and generator as a function of epoch # during training, these values keep fluctuating, suggesting there is no mode collapse as both neural networks get stuck in a local minimum. d) 64 generated slices of synthetic bamboo epidermis images that capture the essential pattern but are not the same as any training images.

We try to learn the hidden pattern of the bamboo epidermis with Generative AI, enabled through DCGAN.^[^ ^16^ ^]^ This algorithm has shown a promising role in understanding the complex structure‐function relationships in complex materials.^[^ ^25^, ^26^ ^]^ It offers significant potential for discovery, such as novo protein structures,^[^ ^27^ ^]^ biomedicine,^[^ ^28^ ^]^ and photonics.^[^ ^29^ ^]^ Our model is enabled by the competing and simultaneous training of the two neuron networks: the generator and discriminator (**Figure** **5**a). The generator tries to generate fake SEM images by starting from noise that is very similar to the original SEM images of the bamboo epidermis of 400 × 400 µm^2^ (each slice in Figure 5b) and the discriminator tries to tell the fake image from the real one correctly. This training continues until the generator can massively produce realistic images that the discriminator cannot fully distinguish from real ones and their loss functions reach equilibrium (Figure 5c). The key output, as a collection of fully synthetic image slices produced by the generator, is shown in Figure 5d as example. They give a very similar particle density and distribution pattern to the original SEM images of the bamboo epidermis. Still, any single synthetic image is not precisely the same as other synthetic ones, nor the original images.

**Figure 6:**
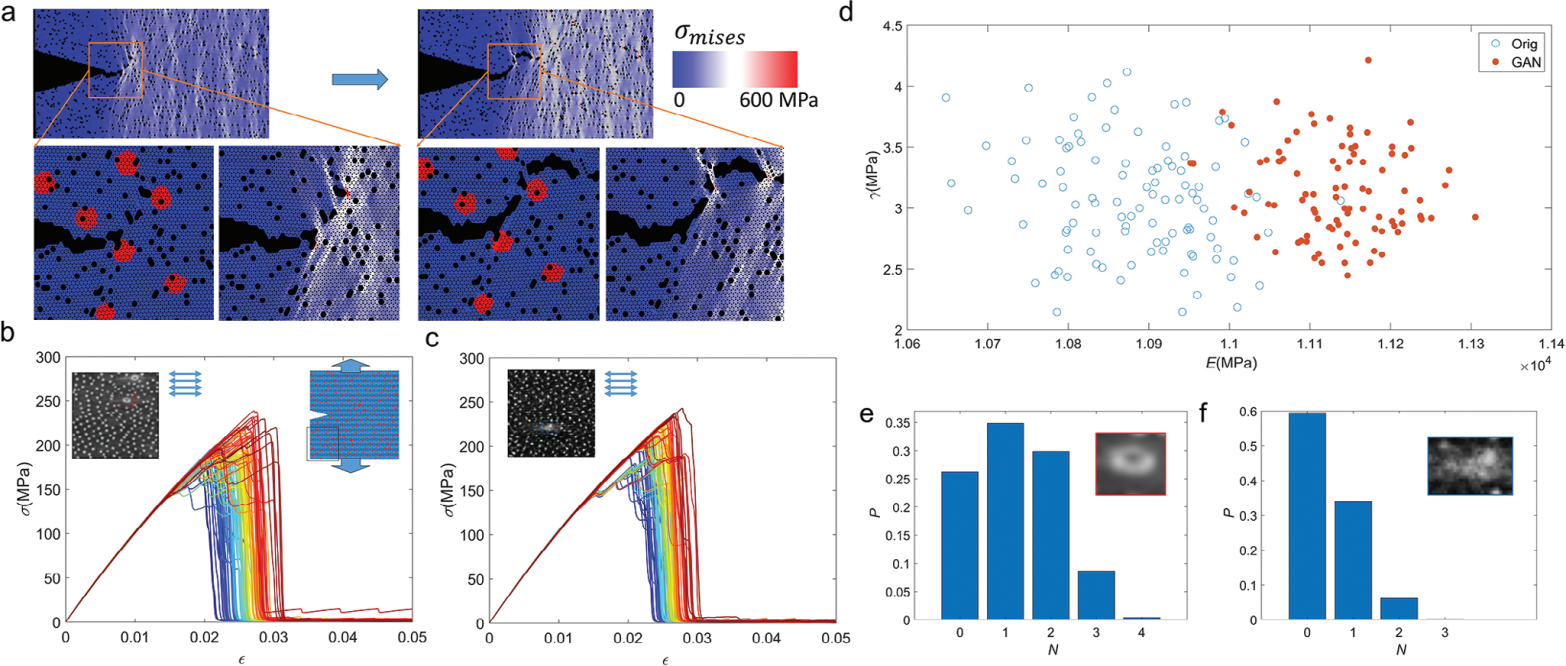
Fracture tests on mechanical models based on original bamboo epidermis images and generated ones. a) Simulation snapshots of the failure process of a bamboo epidermis model with 5% porous defects, with a zoom‐in image of the material composition and von Mises stress near the crack tip. b) Schematic of the fracture test by applying uniaxial tensile stress (σ) on original bamboo‐image‐based samples with a pre‐existing crack (20% of sample length). The mechanical response of the one hundred samples is given by the σ − ε curves below. c) Schematic of the fracture test by applying uniaxial tensile stress (σ) on synthetic‐image‐based samples obtained from the trained DCGAN model with a pre‐existing crack and the σ − ε curves of one hundred samples. d) An Ashby plot summarizes the fracture toughness and elastic modulus as obtained from the test curves of the two test groups. It is shown that while the fracture toughness of the two groups of samples is similar, the elastic modulus given by synthetic samples is higher than the original ones. Histogram of the number of stomata in e) original samples and, f) the synthetic ones. It is shown that DCGAN underestimates the number of stomata in each sample, which explains the higher elastic modulus obtained.

The DCGAN model provides a more effective data augmentation solution, which massively generates new particle distributions without taking additional SEM images. It can be used toward bio‐inspired designs with variety in structure, function, and authentic appearance as their natural counterparts with anisotropic and non‐periodicity. To effectively validate that the synthetic images correspond to a similar fracture toughness as the original bamboo samples in the fracture test without significant weakness, we use a simple elastic network (bead‐spring) model of heterogeneous spring stiffness to simulate the deformation and fracture of 100 randomly selected original and synthetic bamboo epidermis models in a Large‐scale Atomic/Molecular Massively Parallel Simulator (LAMMPS, detailed method and parameters in Table S2 and Table S3, Supporting Information).^[^ ^30^ ^]^ This model enables us to study the bamboo epidermis with cracks and defects and simulate their mechanical response in mechanical loading (**Figure** **6**a, comparing it to pure cellulose in Figure S5, Supporting Information). It is shown in Figure 6a that the silica particles within the bamboo epidermis help it to arrest the crack, deflect its direction, and diffuse the von Mises stress from the crack tip to the large region ahead of it. These simulation results are summarized by the stress‐strain curves given in Figure 6a,b. These curves are statistically similar, although one group is obtained from models based on the original SEM image, and the other group is obtained from models based on the synthetic images. Some saw tooth‐like features before and after the ultimate stress, corresponding to the deflection of crack propagation (Figure S4, Supporting Information) and bridging, shown in both groups. To validate that the crack deflection because of the silica particles can increase the strength and toughness of the bamboo epidermis, we also run the simulations by considering 5% point porosity to account the structural defects such as stomata and heterogeneity. The fracture results, as summarized in Figure S5 (Supporting Information), show that the strength and toughness of the composite (152.7 ± 10.0 MPa, 2.33 ± 0.26 MJ m^−3^, Figure S5c, Supporting Information) are higher than pure cellulose (140.2 ± 14.2 MPa, 2.20 ± 0.28 MJ m^−3^, Figure S5b, Supporting Information). Again, it is shown that the silica particles (Figure S5e, Supporting Information) can deflect the crack path and diffuse the stress concentration at the crack tip, leading to higher strength and toughness than the pure cellulose matrix.

Moreover, the Ashby plots of the elastic modulus (*E*) and fracture toughness (γ) of the original and synthetic models have a large overlapping region, as shown in Figure 6c. The mean fracture toughness is very similar between the two groups. An independent *t*‐test is conducted to compare the two sets of fracture toughness and shows no significant difference (*p‐value* = 0.26, df = 198) between them at the 0.05 significance level. We find that the synthetic models have a slightly higher elastic modulus (≈2%). All the input images for training the DCGAN model have consistent fiber orientation. The mechanical loading is applied perpendicular to the fiber direction so the different loading curves are not caused by the anisotropy. By comparing the structures, we find that the different stiffness is probably caused by the underprediction of the number of stomata within the synthetic samples, as given by the histograms in Figure 6d and e: while most original image slices contain 1–2 stomata defects, most synthetic image slices only contain 0–1 stomata. The stomata are much larger and will not be recognized as rigid silica particles. Instead, it will be merged to the pure cellulose matrix during the modeling process. Thus, a lower stomata number yields a stiffer composite. This observation indicates a potential limitation of the DCGAN as it can extract patterns hidden within frequently appearing structures but is not intelligent enough to extract very rare features.

## 3 Conclusion

By analyzing the microscopic structure of the bamboo epidermis, we reveal that the material is a composite made of relatively soft cellulose matrix and rigid silica particles of anisotropic distribution orders in different material directions. The particles are highly ordered in the direction perpendicular to the bundles of bamboo fibers, and the dense regions distribute along the junctions between the fiber bundles. Since fiber splitting is the primary mode governing bamboo failure in different loading conditions (e.g., buckling, bending, torsion), the silica particles along the fiber junctions effectively enhance the fracture toughness and prevent cracks from propagation. Moreover, by performing mechanical tests on 3D printed samples and numerical models strictly following the geometry obtained from the SEM images, we find that the less ordered particle distribution along the fibers is beneficial in causing crack deflection and bridging for additional energy dissipation. The results explain the complex crack pathway for the microscopic failure of the bamboo epidermis in SEM images. They provide microscopic mechanisms to the experimental observations as the bamboo plywood with epidermis is tougher than the ones without,^[^ ^11^ ^]^ suggesting the critical mechanical enhancement role that the epidermis plays.

In this study, we develop a unique combination of integrating AI, numerical simulation, and 3D printing to rationally mimic the bamboo epidermis, learn the hidden structure‐mechanics relationships, and validate the bioinspired designs in tests. We find that instead of direct mimicking, generative AI provides a powerful tool to extract the hidden pattern within the bamboo epidermis and produce synthetic structures accordingly. This method can be applied to many biological materials for their structure augmentation, helping to extract their structure‐function relationships. Compared to direct copying, AI is more intelligent in extracting essential information from large datasets and is thus suitable for studying biocomposites of complex structures and interfaces between different material phases. It reduces the effort needed to acquire the representative structure and necessary position optimization near the periodic boundary to minimize the discontinuity for assembly associated with apparent mechanical weakness. The generated structures are of different mechanical functions but uniform composition, providing candidates for mapping different regions of a mechanical part that experiences heterogeneous stress distribution in loading. For this purpose, using our DCGAN model to massively generate synthetic bamboo structures, examine their elastic matrix, and explore the latent space for optimal designs will be convenient. Some limitations of the model (e.g., handling high‐resolution images and extracting rare features) should be overcome by combining another DCGAN model trained by much larger samples that make the features more frequent with a clear pattern or using image‐based AI tools that can directly handle high‐resolution SEM images.^[^ ^31^ ^]^ The beginning is to observe particle distribution and its connection to the mechanical advantage of the bamboo epidermis. Further, we can use quantitative structural analysis (i.e., PDF) and effective data augmentation (i.e., DCGAN) to generate particle distribution structures corresponding to different anisotropy levels massively. Figuring out the distribution‐mechanics relationship will require more quantitative simulations, but supervised machine‐learning algorithms can accelerate this process. This knowledge will help us design particle‐reinforced composites, and we can always test the performance of the designs with a 3D printer.

This insight expands the design space of particle‐reinforced composites for enhanced toughness beyond tuning conventional parameters, offering advantages in industries where mechanical reliability is critical. For example, in the aerospace and automotive sectors, these composites could be used to develop mechanically durable composites, improving the safety of engineering designs. In civil engineering, particle‐optimized materials can be applied in impact‐resistant structures, such as protective barriers or earthquake‐resistant building elements. Biomedical applications may benefit from these advancements in designing tough yet flexible implants and prosthetics that mimic the mechanical behavior of natural tissues. Furthermore, this design principle could contribute to their durability in sports equipment and wearable technologies. By expanding the design space with bamboo‐epidermis mimicking configurations, this work unlocks the potential to tailor material functions through a new dimension that will enable composites for applications that demand a precise balance between toughness, modulus, and other functional properties.

Bamboo, a rapid‐growing and renewable plant offers significant biological and economic value, making it an essential resource for future sustainable cities. For example, its unique growth mode and adaptability to various climates make it suitable for wide distribution in towns and local communities for carbon sequestration.^[^ ^32^ ^]^ Its complex root system helps prevent soil erosion and improve soil health for environmental improvements. Its natural strength and flexibility make it an excellent alternative to traditional timber and even metals for building façade, scaffolding, and many manufacturing applications. The knowledge of the microscopic structure‐function relationship of the bamboo epidermis can be combined with the large‐scale behavior of bamboo culm to understand the connection from the initiation of mechanical cracks to its continuum‐scale mechanical buckling and splitting failure in extreme loading, which is crucial to improve the durability of bamboo products. The knowledge learned from bamboo epidermis can also inspire the composite design with a precise definition of the particle distribution, thus impacting sustainable civil developments and composite manufacturing technologies.

## 4 Experimental Section

### PDF of Particle Distribution

The PDF function of particle distribution, giving the probability density of finding a neighboring particle at a certain distance (*d*) from the center particle, is obtained via

(1) $$\begin{array}{ccc} PDF\;\left(d\right) & & =\frac{1}{2\pi dN\cdot \Delta r}\;\sum_{i}\sum_{j}f\left(\left|r_{ij}-d\right|-\frac{\Delta r}{2}\right)\\ & & \cdot f\left(\left|\left|arctg\left(\vec{r_{ij}}\right)\right|-\varphi_{0}\right|-\frac{\Delta \varphi }{2}\right) \end{array}$$

where Δ*r*  =  2 µm is the bin size, *d* is in the range Δ*r*/2<*d*<100 µm, *N* is the total particle number within the sample, *r_ij_* is the distance between two particles of indices *i* and *j*, and *f(a)* is a cutoff function with *f(a)* = 1 for a<0 and *f(a)* = 0 for a>0. The second *f* function is for statistical analysis of the PDF function along a specific direction, where *arctg* function returns the angle direction of the vector $\vec{r_{ij}}$, φ_0_ for the preferred direction with $\varphi_{0}=\frac{\pi }{2}$ for the direction along the fibers and φ_0_ =  0 for the direction perpendicular to fibers (for samples given by their SEM images in Figure S1a, Supporting Information) and Δφ for the preferred range of analysis with Δφ ≥ π for simple no‐directional preference (i.e., PDF for the entire sample) and Δφ < π for only counting the particles distributed within $\varphi_{0}\pm \frac{\Delta \varphi }{2}$ that of a center particle. For directional PDF analysis, Δφ  =  π/3 was generally taken.

### Deviation of Particle Distribution Between Samples

The PDF function of silica particle distribution obtained from the original bamboo SEM images as *PDF_orig_* (*d*) by using Equation (1) was computed. For samples composed of a periodic assembly of representative unit cells, the deviation of its distribution function *PDF_tile_* from *PDF_orig_* was computed to quantify the structural difference via

(2) $$\mathrm{SD}/\;\rho_{m}=\frac{\sqrt{\bar{{\left(PDF_{orig}-PDF_{tile}\right)}^{2}}}}{\rho_{m}}\;$$

where ρ_*m*_ is the mean density of particles within the bamboo epidermis. Its value can be estimated by ρ_*m*_ =  *N*/*L_x_*/*L_y_*, where *L_x_* and *L_y_* are the physical dimensions of a rectangular SEM image of a sample, and *N* is the total particle number. The deviation was computed to ensure that the pattern built from a unit cell closely resembles the original bamboo epidermis. PDF gave the statistical feature of the distribution of neighbors from a center particle, disregarding the local missing data. The relationship between a unit cell area and the mean deviation value for all the unit cells of the same size were plotted. The curve was fitted using an exponentially decaying function $\bar{\mathrm{SD}}/\;\rho_{m}=ae^{-\frac{A_{tile}}{A_{0}}}+b$ where *a*, *b*, and *A*_0_ are fitting parameters with *A*_0_ for the cell area of a balance between patterns with a low $\bar{\mathrm{SD}}/\rho_{m}$ value and small unit cell sizes. Accordingly, unite cells of 40 × 40 µm^2^ (square), 60 × 120 µm^2^ (rectangle of 1:2 aspect ratio), 30 × 90 µm^2^ (rectangle of 1:3 aspect ratio), and 45 × 45 µm^2^ (rhombus or a rotated square) are selected. These unit cells were periodically arranged and tightly packed to reproduce the distribution pattern of silica particles for 3D printing and mechanical testing, as the workflow schematically shows in Figure S3 (Supporting Information).

### 3D Printing of Bamboo‐Epidermis‐Inspired Composite Samples

Samples were designed on AutoCAD using the coordinates of the silica particles. Some complications included slight variations in the size and shape of each silica particle. For simplicity, the particles were designed to be cylindrical, with a constant diameter of 10 µm for its cross‐section. Some smaller, closely spaced particles with overlapping were manually removed according to the SEM image. These operations led to minor differences in the total area of silica particles, which was kept relatively constant at ≈10% of the total sample area. The flexible matrix material rounded the rigid cylinders. To include the structural defects given by stomata, 12 elliptical void defects of 42.2 µm diameter and 10.5 µm along the long and short axis, respectively was introduced. These voids took 5% of the total sample area. A high‐resolution and multi‐material 3D printer, Objet260, made by Stratasys was used, to carry out the samples by uniformly scaling all the structural features 95 times. The 3D printing sample size was 38 × 38 × 1.9 mm^3^ with 1.9 mm as the sample thickness, corresponding to the range of 400 × 400 µm^2^ of the bamboo epidermis with 20 µm for the epidermis thickness.^[^ ^33^ ^]^ The combination of a rigid material, Digital ABS Plus (Young's modulus of 963 MPa, strength of 30 MPa and toughness of 10.5 MPa), and a soft material, Agilus30 (Young's modulus of 0.383 MPa, strength of 0.7 MPa and toughness of 0.56 MPa), was used to print the silica particles and matrix phase of the bamboo epidermis model, respectively. Two rectangular blocks of 38 × 12 × 1.9 mm^3^ were added made of the rigid material to each model's upper and lower boundaries for printing to ensure they could be held for the loading test. The deformation and failure process of the sample is given by snapshots in Figure 4a.

### DCGAN for the Synthetic Image of Bamboo Epidermis

Deep Convolutional Generative Adversarial Networks (DCGAN) was used by including a set of constraints on the architecture of GAN.^[^ ^16^ ^]^ implemented via PyTorch. This algorithm enabled the training of models that learn from the distribution of silica particles in the bamboo epidermis within SEM images and generated new silica distribution designs. Matlab was used to recognize the scale bar of different SEM images and crop sub‐images corresponding to a uniform physical dimension of 400 × 400 µm^2^ of a uniform resolution (128 × 128) for inputs. This model uses a generator to create synthetic images starting from a random noise vector and 6 transposed convolutional layers to gradually increase the spatial resolution of the input to generate a final output image. It is noted that the image resolution limit of classical DCGAN (32 × 32 or 64 × 64) was increased by increasing kernel size and number of transpose layers. Further increasing the image resolution led to mode collapse when the discriminator got stuck in a local minimum and does not found its way out by optimizing its parameters. A collapsed model kept repeating the same output and refrained from further training. The other part of the model was a discriminator to differentiate between real and fake images with 6 convolutional layers to gradually reduce the spatial resolution and extract features from the input image. Leaky ReLU activation was used after each convolutional layer, with the final layer using a sigmoid activation function to output a probability score representing whether the input was real or fake. For each round the generator and discriminator played a min‐max game by learning from a portion of the training data (batch size of 64): the discriminator tried to minimize the error of classifying images (*x_i_* and *G*(*z_i_*) for images from the training set and generated, respectively) as either real (i.e., from the dataset) or fake (i.e., produced by the generator); the generator tried to generate images from noise (*G*(*z_i_*)) and maximize the error of the discriminator. The logarithmic loss function values of the generator and discriminator were computed via, respectively, where *m* is the number of images within a batch, *D*(*x_i_*) is the probability that the discriminator assigns to the real images *x_i_* with returns being real, and *G*(*z_i_*) is an image generated by the generator from a noise vector *z_i_*. In case a logarithmic function had zero input, a small positive ε was added to the argument of the logarithm to prevent it from being zero. Using batch averaging helped to provide stable gradient estimates during training. One complete pass through the entire training data (in total 500 images) corresponded to an epoch as the *Loss_D_* and *Loss_G_* values were recorded (Figure 5c). Both of them were simultaneously trained until the generator could massively produce realistic images that the discriminator cannot fully distinguish from real ones when equilibrium was reached. Using the generative model to generate the new distribution pattern, the coordinates of the particle centers were extracted with Hough's transform, use the coordinates to obtain PDF of the generated patterns. The PDFs of all the images were compared to ensure the difference between the PDFs of any generated image and any training image was in a similar order of magnitude to the difference of any pairs of training images. The directional PDF was also checked to ensure the generated structures significantly differ in direction along the fibers and perpendicular to the fibers for the anisotropy feature.

(3) $$Loss_{D}=-\frac{1}{m}\sum_{i=1}^{m}\left[\log\left(D\left(x_{i}\right)\right)+\log\left(1-D\left(G\left(z_{i}\right)\right)\right)\right]$$

(4) $$Loss_{G}=-\frac{1}{m}\sum_{i=1}^{m}\log\left(1-D\left(G\left(z_{i}\right)\right)\right)$$

## Conflict of Interest

The authors declare no conflict of interest.

## Author Contributions

Z.Q. performed conceptualization, supervision, research protocol, results analysis, and validation, and wrote, reviewed, and edited the final manuscript. A.P.D. performed data cleaning and preparation, 3D printing and mechanical testing, results analysis, and reporting.

## Supporting information



Supporting Information

## Data Availability

The data that support the findings of this study are available in the supplementary material of this article.
