# Supplementary material for: The Structure‐Mechanics Relationship of Bamboo‐Epidermis and Inspired Composite Design by Artificial Intelligence
Source: Adv Mater. 2024 Dec 27;37(22):2414970. doi: 10.1002/adma.202414970 (PMC12138844; doi:10.1002/adma.202414970)
Supplement: Supplementary file 1 — Supporting Information [file ADMA-37-2414970-s001.docx]

**Supporting Information**

**The Structure-mechanics Relationship of Bamboo-epidermis and Inspired Composite Design by Artificial Intelligence**

Zhao Qin^1,2,3^*, Aymeric Pierre Destree^1,2^

**Supporting Information 1: Supporting Experimental Methods**

***Extraction of Silica Particles from SEM images of Bamboo Epidermis***

The surface of *Pseudosasa amabilis* bamboo epidermis samples was cut and polished with a grinder and polishing papers with grit sizes up to 2000. The images were taken under a TESCAN SEM with details given in the previous work ^[10]^. We took many SEM images (Fig. 2A & Fig. S1A) to reveal that most of the particles on the polished surface is concentrated in the shape of circular particles. To understand the composition and accurately measure the diameter of the embedded particles, we analyzed the interface between particle and matrix, which is given by the BSE image of the carbon and silicone distribution (Fig. 1 & Fig. S2A-C). Using Hough transformation with Matlab we automatically identify all the circle centers in the image and statistically obtain the elemental distribution around all the centers (Fig. 2B & Fig. S2D-E). It is clearly shown that the particles embedded in bamboo are mainly composed of silica with a small amount of cellulose at the interface.

***FFT of particle distribution and fiber orientation***

After extracting the position and size of silica particles, we apply FFT in Matlab to understand their overall distribution. We use the two-dimensional FFT algorithm to convert the distribution to frequency space and then take the absolute value, add 1 to avoid undefined values, and apply a log transformation to visualize the data displayed in log space (Fig. S1C). This operation reduce the huge difference between individual data points and enable to visualize the entire frequency space. The images shows the contrasting zero and non-zero value along the horizontal axis than other directions, suggesting the more significant periodicity of the particles that is coincidentally perpendicular to the bamboo fiber direction.

***Mechanical loading test of 3D printed samples***

The tensile tests were carried out at room temperature with a standard Instron machine (Instron 5966, 10 kN load cell and 1 kN pressure-drive grips). In each test, the upper and lower boundaries of the sample were clamped to a crosshead that moved at a constant rate of 0.1 mm/s. The upper grips connected to a force transducer for measuring the reaction force. The applied tensile force and the total displacement were scanned at 50 Hz and saved to the connected computer. The force is normalized by the cross-section area of the sample to obtain the stress (*σ*) and the total displacement is normalized by the initial length of the sample to obtain the strain (*ε*).

***Numerical modeling of bamboo epidermis for fracture tests***

We use a regular triangular bead-spring network to model the mechanical behavior of silica and cellulose matrix and their combination as the bamboo epidermis in tensile loading tests. Each mass bead bonds to its six neighbor beads accounts for the material density. Using the relationship between the deformation energy of the network and the continue material in biaxial tensile, the spring constant *k* as the stiffness of each spring at zero deformation is defined by

$k=\frac{\sqrt{3}}{2}Et$ (4)

where $E$ is the Young’s modulus of different material phases and *t* is the thickness of the material. To incorporate the bond rupture, each spring is modelled by a Morse potential with the deformation energy given by

$U=D\left[ 1-e^{-\alpha(r-a)} \right]^{2}$ (5)

Where *a*=1.306 μm is the equilibrium length of the spring to ensure that is fine enough for the geometry of a single silica particle, *D* and $\alpha$ are two parameters relates to the network strength and stiffness as

$\alpha=\frac{3E}{8\sigma_{c}a}$ (6)

and

$D=\frac{16\sqrt{3}}{9}\sigma_{c}^{2}a^{2}t/E$ (8)

Where $\sigma_{c}$ is the ultimate strength of different material phases with their material properties summarized in Table S2. We use Large-scale Atomic/Molecular Massively Parallel Simulator (LAMMPS ^[28]^) to run the numerical simulation for bamboo epidermis deformation with the unit system of “*micro*”. The numerical values of the two material phases with corresponding units are summarized in Table S3. We run a quasi-static loading test by mapping the deformation to all beads, fixing the position of beads within the lower and upper boundaries, and summing the total reaction force on all the beads within each loading step and obtain the *σ−ε* relationship accordingly.

*128×128-pixel-image-based DCGAN training Python code*

| ##PyTorch implementation of DCGAN introduced in the paper: [Unsupervised Representation ##Learning with Deep Convolutional  ##Generative Adversarial Networks](https://arxiv.org/abs/1511.06434), Alec Radford, Luke Metz, ##Soumith Chintala.  ##Developed based on PyTorch Implementation of DCGAN trained on the CelebA dataset  ##(https://github.com/Natsu6767/DCGAN-PyTorch) dcgan.py  ##128*128 DCGAN  import torch  import torch.nn as nn  import torch.nn.functional as F  def weights_init(w):  """  Initializes the weights of the layer, w.  """  classname = w.__class__.__name__  if classname.find('conv') != -1:  nn.init.normal_(w.weight.data, 0.0, 0.02)  elif classname.find('bn') != -1:  nn.init.normal_(w.weight.data, 1.0, 0.02)  nn.init.constant_(w.bias.data, 0)  # Define the Generator Network  class Generator(nn.Module):  def __init__(self, params):  super().__init__()  # Input is the latent vector Z.  self.tconv1 = nn.ConvTranspose2d(params['nz'], params['ngf']*16,  kernel_size=4, stride=1, padding=0, bias=False)  self.bn1 = nn.BatchNorm2d(params['ngf']*16)  # Input Dimension: (ngf*8) x 4 x 4  self.tconv2 = nn.ConvTranspose2d(params['ngf']*16, params['ngf']*8,  4, 2, 1, bias=False)  self.bn2 = nn.BatchNorm2d(params['ngf']*8)  # Input Dimension: (ngf*4) x 8 x 8  self.tconv3 = nn.ConvTranspose2d(params['ngf']*8, params['ngf']*4,  4, 2, 1, bias=False)  self.bn3 = nn.BatchNorm2d(params['ngf']*4)  # Input Dimension: (ngf*2) x 16 x 16  self.tconv4 = nn.ConvTranspose2d(params['ngf']*4, params['ngf']*2,  4, 2, 1, bias=False)  self.bn4 = nn.BatchNorm2d(params['ngf']*2)    # Input Dimension: (ngf) * 32 * 32  self.tconv5 = nn.ConvTranspose2d(params['ngf']*2, params['ngf'],  4, 2, 1, bias=False)  self.bn5 = nn.BatchNorm2d(params['ngf'])  # Input Dimension: (ngf) * 64 * 64  self.tconv6 = nn.ConvTranspose2d(params['ngf'], params['nc'],  4, 2, 1, bias=False)  #Output Dimension: (nc) x 128 x 128  def forward(self, x):  x = F.leaky_relu(self.bn1(self.tconv1(x)),0.2,True)  x = F.leaky_relu(self.bn2(self.tconv2(x)),0.2,True)  x = F.leaky_relu(self.bn3(self.tconv3(x)),0.2,True)  x = F.leaky_relu(self.bn4(self.tconv4(x)),0.2,True)  x = F.leaky_relu(self.bn5(self.tconv5(x)),0.2,True)  x = F.sigmoid(self.tconv6(x))  return x  # Define the Discriminator Network  class Discriminator(nn.Module):  def __init__(self, params):  super().__init__()  # Input Dimension: (nc) x 128 x 128  self.conv1 = nn.Conv2d(params['nc'], params['ndf'],  4, 2, 1, bias=False)  # Input Dimension: (ndf) x 64 x 64  self.conv2 = nn.Conv2d(params['ndf'], params['ndf']*2,  4, 2, 1, bias=False)  self.bn2 = nn.BatchNorm2d(params['ndf']*2)  # Input Dimension: (ndf*2) x 32 x 32  self.conv3 = nn.Conv2d(params['ndf']*2, params['ndf']*4,  4, 2, 1, bias=False)  self.bn3 = nn.BatchNorm2d(params['ndf']*4)  # Input Dimension: (ndf*4) x 16 x 16  self.conv4 = nn.Conv2d(params['ndf']*4, params['ndf']*8,  4, 2, 1, bias=False)  self.bn4 = nn.BatchNorm2d(params['ndf']*8)  # Input Dimension: (ndf*4) x 8 x 8  self.conv5 = nn.Conv2d(params['ndf']*8, params['ndf']*16,  4, 2, 1, bias=False)  self.bn5 = nn.BatchNorm2d(params['ndf']*16)  # Input Dimension: (ndf*8) x 4 x 4  self.conv6 = nn.Conv2d(params['ndf']*16, 1, 4, 1, 0, bias=False)  def forward(self, x):  x = F.leaky_relu(self.conv1(x), 0.2, True)  x = F.leaky_relu(self.bn2(self.conv2(x)), 0.2, True)  x = F.leaky_relu(self.bn3(self.conv3(x)), 0.2, True)  x = F.leaky_relu(self.bn4(self.conv4(x)), 0.2, True)  x = F.leaky_relu(self.bn5(self.conv5(x)), 0.2, True)  x = F.sigmoid(self.conv6(x))  return x |
| --- |

**Supporting Information 2: Supporting Figures**





**Figure S1. SEM images of bamboo epidermis and their corresponding image-based analysis results. a.** SEM image of bamboo epidermis, with silica particles given by a lighter color, arrows in the image suggest the fiber direction. **b.** The image compose of only silica particles as extracted from the SEM image by Hough transformation. **c.** Outcome of 2D-FFT of images in panel **b**. **d.** PDF of particle distribution in the entire field. **e.** The PDF of particle distribution only along the fiber direction ($\frac{\pi}{2}-\frac{\pi}{6}<\varphi<\frac{\pi}{2}+\frac{\pi}{6}$, as defined in Eq. 1). **f.** The PDF of particle distribution is only vertical to fiber direction ($-\frac{\pi}{6}<\varphi<\frac{\pi}{6}$).





**Figure S2. Back-scattered electron (BSE) image of SEM for element distribution in the bamboo epidermis. a.** The overlay of silicon and carbon distribution. **b.** The carbon distribution. **c.** The silicon distribution. **d.** Schematic of the material distribution around the center of a silica particle. **e.** The statistical result of transition from silica to amorphous and then cellulose from the particle center (*r*=0) to far away from silica particles, according to the RGB pixel color depth (G for cellulose and R for silica) in BSE images.


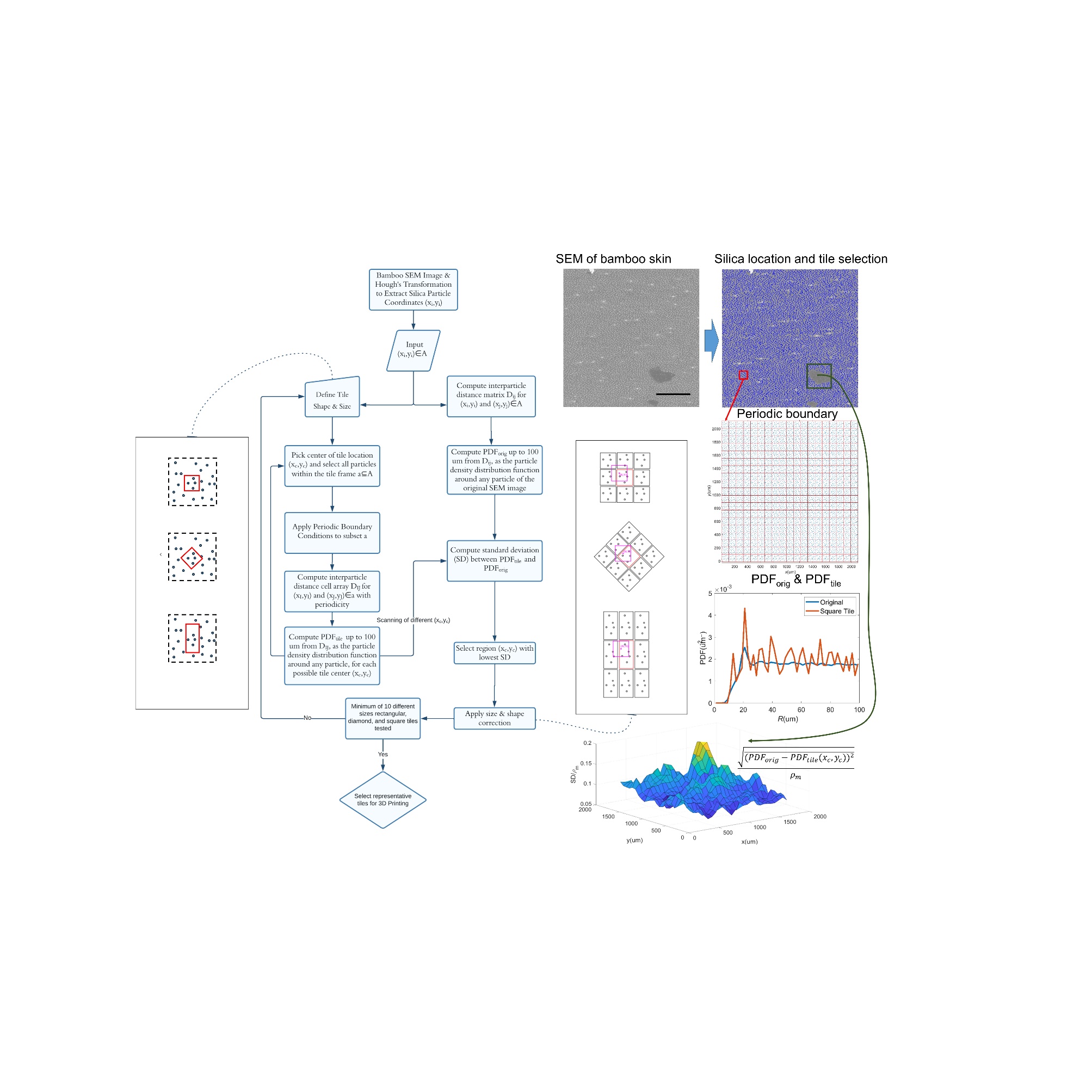


**Figure S3. Schematic workflow of extracting small unit cells from bamboo SEM image that reproduce the PDF distribution as found in original large samples.**





**Figure S4. The mechanical response of the elastic network model of bamboo epidermis in tensile loading simulations. a.** The *σ−ε* curves of pure cellulose, pure silica and bamboo models in quasi-static tensile loading tests. **b.** The simulation snapshots taken at different deformation stages (i~iv) corresponding to panel a. **c.** The *σ−ε* curves of pure cellulose, pure silica and bamboo models with a pre-existing crack of 20% the total sample width in quasi-static tensile loading tests. **d.** The simulation snapshots taken at different deformation stages (i~iv) corresponding to panel c. Scale bars: 60 μm.





**Figure S5. Simulation result of the fracture of pure cellulose and bamboo epidermis in mechanical loading.** The simulation models are the same dimension as in Fig. S4d but of 5% porosity. **a.** The simulation snapshots of the pure cellulose near the crack tip. The elastic network is colored according to the von Mises stress with the color legend given at the bottom of the figure. **b.** The stress-strain relationship of four pure cellulose models. Each cellulose model is used to generate five bamboo epidermis models with the coordinate of the silica taken from the bamboo SEM pictures (**Fig. S1a_v**), that correspond to 20 stress-strain curves as given in **c. d.** The ultimate strength ($\sigma_{C}$) and fracture toughness ($\gamma$) of the bamboo composite and pure cellulose models, and the bamboo models shows higher strength and toughness than the cellulose models. **e.** The fracture process of a bamboo composite model, with blue for the cellulose matrix and red for the silica particles and each structure correspond to the von Mises stress plot in **f.**

**Supporting Information 3: Supporting Tables**

**Table S1.** The mechanical properties of different 3D printed models as measured from the outcome of tensile tests.

| **Sample** | **Young’s modulus, *E* (MPa)** | **Toughness, *U* (MJ/m3)** | **Difference with** $\boldsymbol{E}_{\boldsymbol{original}}$ **(%)** | **Difference with** $\boldsymbol{U}_{\boldsymbol{original}}$ **(%)** | **Volume of particle (%)** |
| --- | --- | --- | --- | --- | --- |
| Original | 5.87±1.40 | 0.404±0.036 | 0 | 0 | 13.11 |
| Square | 5.09±1.11 | 0.397±0.026 | -13.33 | -1.81 | 12.67 |
| Diamond | 6.08±0.39 | 0.408±0.026 | 3.58 | 0.87 | 14.10 |
| Rectangle | 5.57±0.31 | 0.390±0.013 | -5.16 | -3.54 | 11.49 |
| Random | 6.06±0.25 | 0.362±0.031 | 3.17 | -10.46 | 13.35 |

**Table S2:** The basic mechanical properties of the two material phases (silica and cellulose), as obtained from experimental measurements, in SI unit.

| **Parameter** | **Silica** | **Cellulose** |
| --- | --- | --- |
| Density (g/cm^3^) | 2.3 | 1.6 |
| Young’s modulus (MPa) | 72000 | 10700 |
| Ultimate strength (MPa) | 2500 | 600 |
| Poisson’s ratio | 0.2 | 0.23 |

**Table S3:** Numerical values of the potential parameters of the two material phases (silica and cellulose) for coarse-grained MD simulations, in the unit system *Micro* of LAMMPS simulation package.

| **Parameter** | **Silica** | **Cellulose** |
| --- | --- | --- |
| Material thickness *t* (μm) | 1 | 1 |
| Young’s modulus *E* (pg/(μm*$s^{2})$) | $7.2\times{10}^{7}$ | $1.07\times{10}^{7}$ |
| Ultimate strength $\sigma_{c}$ (pg/(μm*$s^{2})$) | 2500000 | 600000 |
| Bond stiffness *k* (pg/$s^{2}$) | $3.12\times{10}^{7}$ | $4.63\times{10}^{6}$ |
| Bond length *a* (μm) | 1.306 | 1.306 |
| Bond parameter $\alpha$ (1/μm) | 8.270 | 5.121 |
| Bond energy $D$ (pg*μm^2^/$s^{2}$) | $4.56\times{10}^{5}$ | $1.77\times{10}^{5}$ |
